# Supplementary material for: Trends and predictors of in-hospital mortality among babies with hypoxic ischaemic encephalopathy at a tertiary hospital in Nigeria: A retrospective cohort study
Source: PLoS One. 2021 Apr 26;16(4):e0250633. doi: 10.1371/journal.pone.0250633 (PMC8075215; doi:10.1371/journal.pone.0250633)
Supplement: S1 Table — (DOCX) [file pone.0250633.s001.docx]

**S1 Table. Trends in admission, asphyxia and fatality.**

| **Year** | **Admissions** | **HIE cases** | **HIE cases per 100 admissions** | **HIE cases per thousand-admissions** | **HIE mortality** | **Case Fatality rate(%)** |
| --- | --- | --- | --- | --- | --- | --- |
| 2015 | 687 | 63 | 9.17 | 91.70 | 11 | 17.46 |
| 2016 | 900 | 68 | 7.56 | 75.56 | 18 | 26.47 |
| 2017 | 944 | 49 | 5.19 | 51.91 | 12 | 24.49 |
| 2018 | 861 | 46 | 5.34 | 53.43 | 12 | 26.09 |
| 2019 | 1007 | 86 | 8.54 | 85.40 | 26 | 30.23 |
