# Supplementary material for: Trends and predictors of in-hospital mortality among babies with hypoxic ischaemic encephalopathy at a tertiary hospital in Nigeria: A retrospective cohort study
Source: PLoS One. 2021 Apr 26;16(4):e0250633. doi: 10.1371/journal.pone.0250633 (PMC8075215; doi:10.1371/journal.pone.0250633)
Supplement: S2 Table — (DOCX) [file pone.0250633.s002.docx]

**S2 Table. Life table of the survival experience of the babies with HIE.**

|  | Beg. | Net | Survivor | Std. |  |  |
| --- | --- | --- | --- | --- | --- | --- |
| Time | Total | Fail | Lost | Function | Error | [95% Conf. Int.] |
|  |  |  |  |  |  |  |
| .0417 | 312 | 3 | 0 | 0.9904 | 0.0055 | 0.9705 0.9969 |
| .0833 | 309 | 2 | 0 | 0.9840 | 0.0071 | 0.9619 0.9933 |
| .5 | 307 | 5 | 0 | 0.9679 | 0.0100 | 0.9413 0.9826 |
| .8333 | 302 | 1 | 0 | 0.9647 | 0.0104 | 0.9372 0.9803 |
| .9167 | 301 | 0 | 1 | 0.9647 | 0.0104 | 0.9372 0.9803 |
| .9583 | 300 | 1 | 1 | 0.9615 | 0.0109 | 0.9332 0.9780 |
| 1 | 298 | 32 | 1 | 0.8583 | 0.0198 | 0.8143 0.8925 |
| 1.5 | 265 | 1 | 0 | 0.8550 | 0.0200 | 0.8107 0.8897 |
| 2 | 264 | 15 | 1 | 0.8065 | 0.0224 | 0.7579 0.8463 |
| 3 | 248 | 7 | 2 | 0.7837 | 0.0234 | 0.7335 0.8255 |
| 4 | 239 | 1 | 1 | 0.7804 | 0.0235 | 0.7300 0.8225 |
| 5 | 237 | 3 | 14 | 0.7705 | 0.0239 | 0.7195 0.8135 |
| 6 | 220 | 2 | 5 | 0.7635 | 0.0242 | 0.7120 0.8071 |
| 7 | 213 | 2 | 24 | 0.7564 | 0.0245 | 0.7043 0.8006 |
| 8 | 187 | 1 | 10 | 0.7523 | 0.0247 | 0.6999 0.7969 |
| 9 | 176 | 0 | 19 | 0.7523 | 0.0247 | 0.6999 0.7969 |
| 10 | 157 | 1 | 70 | 0.7475 | 0.0250 | 0.6945 0.7927 |
| 11 | 86 | 0 | 6 | 0.7475 | 0.0250 | 0.6945 0.7927 |
| 12 | 80 | 0 | 14 | 0.7475 | 0.0250 | 0.6945 0.7927 |
| 13 | 66 | 0 | 5 | 0.7475 | 0.0250 | 0.6945 0.7927 |
| 14 | 61 | 0 | 11 | 0.7475 | 0.0250 | 0.6945 0.7927 |
| 15 | 50 | 0 | 16 | 0.7475 | 0.0250 | 0.6945 0.7927 |
| 16 | 34 | 0 | 5 | 0.7475 | 0.0250 | 0.6945 0.7927 |
| 17 | 29 | 0 | 4 | 0.7475 | 0.0250 | 0.6945 0.7927 |
| 18 | 25 | 0 | 5 | 0.7475 | 0.0250 | 0.6945 0.7927 |
| 19 | 20 | 0 | 5 | 0.7475 | 0.0250 | 0.6945 0.7927 |
| 20 | 15 | 0 | 6 | 0.7475 | 0.0250 | 0.6945 0.7927 |
| 22 | 9 | 0 | 4 | 0.7475 | 0.0250 | 0.6945 0.7927 |
| 23 | 5 | 0 | 1 | 0.7475 | 0.0250 | 0.6945 0.7927 |
| 24 | 4 | 2 | 0 | 0.3738 | 0.1873 | 0.0693 0.6957 |
| 27 | 2 | 0 | 1 | 0.3738 | 0.1873 | 0.0693 0.6957 |
| 28 | 1 | 0 | 1 | 0.3738 | 0.1873 | 0.0693 0.6957 |
