# Supplementary material for: Trends and predictors of in-hospital mortality among babies with hypoxic ischaemic encephalopathy at a tertiary hospital in Nigeria: A retrospective cohort study
Source: PLoS One. 2021 Apr 26;16(4):e0250633. doi: 10.1371/journal.pone.0250633 (PMC8075215; doi:10.1371/journal.pone.0250633)
Supplement: Attachment — Submitted filename: Manuscript for Submission- review.docx [file pone.0250633.s003.docx]

**Trends and predictors of in-hospital mortality among babies with hypoxic ischaemic encephalopathy at a tertiary hospital in Nigeria: a retrospective cohort study**

**Beatrice Ezenwa ^1,2*^, Gbenga Olorunfemi ^3,4^, Iretiola Fajolu ^1,2^, Toyin Adeniyi ^2^, Khadijah Oleolo-Ayodeji** **^2^, Blessing Kene-Udemezue ^2^, Joseph A. Olamijulo ^3^, Chinyere Ezeaka ^1,2^**

^1^ Neonatology unit, Department of Paediatrics, College of Medicine University of Lagos, Lagos, Nigeria

^2^ Department of Paediatrics, Lagos University Teaching Hospital, Lagos, Nigeria

^3^ Department of Obstetrics & Gynecology, Lagos University Teaching Hospital, Lagos, Nigeria

^4^Division of Epidemiology and Biostatistics, School of Public Health, University of Witwatersrand, Johannesburg, South Africa

**^*^Corresponding author**

**E**mail: [beatriceezenwa@yahoo.com](mailto:beatriceezenwa@yahoo.com) (BE)

**Abstract**

**Background:** Globally, approximately 9 million neonates develop perinatal asphyxia annually of which about 1.2 million die. Majority of the morbidity and mortality occur in Low and middle-income countries. However, little is known about the current trend in incidence, and the factors affecting mortality from hypoxic ischaemic encephalopathy (HIE), in Nigeria.

Objective: We assessed the trends in incidence and fatality rates and evaluated the predictors of mortality among babies with HIE over a five-year period.

**Methods:** A temporal trend analysis and retrospective cohort study of asphyxiated babies admitted to the neonatal unit of a Nigerian Teaching Hospital was conducted. The socio-demographic and clinical characteristics of the babies and their mothers were extracted from the neonatal unit records. Kaplan-Meir plots and Multivariable Cox proportional hazard ratio was used to evaluate the survival experienced using Stata version 16 (StataCorp USA) statistical software.

**Results:** The median age of the newborns at admission was 26.5 (10 – 53.5) hours and the male to female ratio was 2.1:1. Nearly half (48.1%) were admitted within 24 hours of life and majority (84%) of the infants were out-born. The prevalence and fatality rate of HIE was 7.1% and 25.3% respectively. The annual incidence declined by 1.4% per annum while the annual fatality rate increased by 10.3% per annum from 2015 to 2019.

About 14.1% died within 24 hours of admission. The hazard of death was related to the severity of asphyxia (p=0.002), antenatal booking status of the mother (p=0.01) and place of delivery (p=0.036).

**Conclusio**n: The case fatality rate of HIE is high and increasing. Four-fifth of HIE admissions were outborn and the hazard of death was four-fold higher among the outborn. Community level interventions including skilled birth attendants at delivery, newborn resuscitation trainings for healthcare personnel and capacity building for specialized care should be intensified to reduce the incidence, morbidity and mortality from perinatal asphyxia.

**Introduction**

Hypoxic ischaemic encephalopathy (HIE) is a neurological complication from the inability to establish and sustain respiration at birth in a newborn. It is a major cause of neonatal morbidity and mortality globally. [1-3] Babies who did not initiate or sustain breathing at birth are said to be asphyxiated. Asphyxia causes an impairment of gas exchange that results not only in the deficit of oxygen in the blood but also an excess of carbon dioxide causing acidosis. The acidosis further leads to hypotension and ischemia culminating in hypoxic-ischemic injury. Hypoxic-ischemic brain injury is a major cause of acute mortality and chronic neurologic disability amongst survivors. HIE associated deaths are the fifth most common cause of under-five deaths accounting for 814,000 deaths annually. [2,4] According to World Health Organization, between four to nine million newborns develop birth asphyxia each year of which an estimated 1.2 million die and at least the same number develop severe long-lasting neurological sequelae such as cerebral palsy, epilepsy and cognitive impairments. [5,6,7] In most high-income countries, the incidence of HIE has reduced significantly following improvements in obstetric and neonatal care unlike in lowest and middle-income countries (LMICs). [7,8] In Sub-Saharan Africa and indeed in Nigeria, the real burden of HIE is difficult to determine due to the paucity of data. [9] Aliyu et al [2] reported that HIE contributed as high as 42 million disability-adjusted life years among affected individuals. Newborns that suffer HIE have higher mortality rates and it contributes to high rates of morbidity in neonatal survivors. [10,11] Several factors influence the survival rate in babies that developed HIE. Such factors include the place of birth, level of prenatal care, the cause of asphyxia, gestational age, maternal age, maternal illness, socioeconomic status, availability of resources for neonatal care and time to get to specialized care. [3,5,6] Furthermore, the grade of HIE can also impact on survival. Thus, the Sarnat and Sarnat staging of HIE has been identified as a predictive tool in prognosticating severe perinatal asphyxia. However, this scoring tool requires a certain level of medical expertise. [8,9, 12,13]

Over the last decade, Nigeria embarked on a nationwide training of healthcare providers on strategies to eliminate preventable newborn deaths. [14] Policies and guidelines were established for “essential newborn care”, “helping babies breathe” among others. [14,15] The emphasis was majorly on prevention strategies. Health care providers were mandated and encouraged to undertake the newborn resuscitation and helping babies breathe trainings while mothers were encouraged to register and attend antenatal care and also deliver at facilities with skilled birth attendants. [16] These strategies are expected to reduce perinatal morbidity and mortality and improve newborn survival in Nigeria. However, there is no recent evidence of the impact of the interventions on the trends in HIE in our environment to guide the Government on how to update and improve on current public health intervention strategies. While researchers in Nigeria had utilized Cox proportional analysis to evaluate neonatal mortality, however, appropriate robust cohort analysis were not utilized for analysis of in-hospital mortality of babies with HIE. Therefore, we aimed to evaluate the asphyxia trends and the predictors of immediate in-hospital mortality among babies with HIE at a tertiary care hospital in Nigeria during a five-year period to inform further interventions

**Materials and methods**

This study was a temporal trends analysis and retrospective cohort study of babies admitted to the neonatal units of the Lagos University Teaching Hospital (LUTH) from 1 January 2015 to 31^st^ December, 2019. LUTH is one of the three publicly funded tertiary health institutions in Lagos, South-Western Nigeria. Lagos is a cosmopolitan City with an estimated population of over 14 million residents according to the World Population Review. [17] LUTH also serves the neighboring States and receives referral from primary and secondary health facilities including private health facilities. The neonatal unit of the hospital had 80 cot/ incubator spaces with inborn and outborn sections. The inborn neonatal ward adjoined the labour ward and all caesarean sections and high-risk deliveries were attended by the neonatologists. The admitted newborns with HIE were managed according to the unit protocol for HIE which is majorly supportive care. [13] During the study period, the socio-demographic characteristics, birth history and clinical characteristics of the babies and their mothers were extracted from the records of the neonatal unit admission registry into an excel spreadsheet for analysis. Data collected included: maternal age, parity, mode of delivery, age of infant at admission, sex, gestational age, place of delivery (inborn/outborn), birth weight, duration of admission and outcome (death, or survival). Babies whose mothers delivered in LUTH were classified as “inborn”. Mothers who delivered in LUTH but did not receive antenatal care in LUTH were further classified as “inborn unbooked” to distinguish them from “inborn booked” patients who were patients that their mothers had antenatal care in LUTH and delivered in LUTH. Those babies that were delivered outside LUTH were classified as “Outborn”. The “Outborn” babies were delivered either at the government health care facilities, private health facilities, facilities run by Traditional birth attendants (TBA) or at home before referral to LUTH. As per the protocol of the unit, on the admission of each baby, the severity of HIE was determined based on the Sarnat and Sarnat staging. The HIE severity was classified as mild (Sarnat stage 1), moderate (Sarnat stage 2) and severe (Sarnat stage 3) [12,13].

**Ethical considerations**

This is a retrospective anonymous data collection with no potential ethical breach. However, the data collection process of the database that was utilized has ethical approval from the Health Research and Ethics Committee of LUTH.

**Statistical analysis**

The data was imported into Stata version 16 (StataCorp, Texas USA) statistical software for analysis from excel spreadsheet. Data cleaning and validation was done. Categorical variables were described as frequencies and percentages while continuous variables were presented as mean (± standard deviation) or median (interquartile range) – if not normally distributed.

The annual incidence of HIE was calculated by dividing the annual number of HIE cases by the number of neonatal admissions. The annual case fatality was also calculated by dividing the number of HIE deaths by the annual number of HIE cases multiplied by 100. The incidence and fatality rate were then plotted on graphs and the annual percent change of the trends were calculated from 2015 to 2019. The prevalence (with 95% Confidence interval) of HIE was also calculated. The prevalence (with 95% Confidence interval) of the grades or severity of HIE was also calculated. The association between neonatal and maternal characteristics and severity of HIE was assessed using Pearson’s Chi-square or Fischer’s exact (for categorical variables), one-way analysis of variance (for continuous variables) or Kruskal Wallis test (for non-normally distributed continuous variables).

Time to death was the time-varying variable. Babies that died were coded as 1 while babies that were discharged alive were right-censored and coded as 0. Life tables of the survival experience was produced. Survival experience by various baseline categorical characteristics (sex, severity, inborn/outborn etc) were compared using the Kaplan Meir survival plots and log-rank test. Univariable and multivariable Cox proportional hazard regression modelling was performed with the baseline characteristics as the covariates. Variables with univariable p-value < 0.2 were used to build the multivariable model using backward elimination method. Some variables were selected *a priori*. Crude and adjusted hazard ratio (95% confidence interval) were calculated. Final multivariable Cox proportional hazard model adjusted for HIE severity, gender, weight, age at presentation, type of facility, year of admission. Some variables such as baby’s age, birth weight was handled both as continuous and categorical variable. Two-tailed test of hypothesis was assumed and a P-value <0.5 was set as statistically significant level. Post regression estimation tests such as the Schoenfeld’s test of proportional hazard assumptions was conducted and a p-value >0.05 showed no violation of the assumption of proportional hazard. The variance inflation factor was conducted and a value < 10 shows no collinearity among explanatory variables.

**Results**

**Socio demographic characteristics**

During the 5-year study period spanning January 2015- December 2019, a total of 4399 ill infants were admitted, into the neonatal wards of LUTH. Among these, 312 babies were admitted, on account of HIE. Table 1 shows the Socio-biologic characteristics of infants with HIE. The median age at admission of the infants that suffered HIE was 26.5 (10 – 53.5) hours while the youngest and oldest baby at admission were within 1^st^ hour of life and 24days (576 hours) old, respectively. Nearly half of the babies were admitted within 24 hours of life (48.1%). The male to female ratio was 2.1:1. Majority, (n= 257/312, 84%) of the admitted babies were outborn; and three-fifth, (n=30/49, 61.22%) of the 49 inborn babies, were delivered by unbooked mothers in LUTH. Almost all (n= 296/312, 94.9%) the infants were delivered at term (≥37 weeks) and median weight at admission was 3000g (IQR: 2700 – 3475). Almost half (n= 150/312, 48.08%) of the babies with HIE, were admitted within 24 hours of delivery with a quarter presenting between 24 - 48 hours (n=81/312, 25.96%) and beyond 48 hours (n=81/312, 25.96%), respectively) (Table 1)

**Table 1. Socio-biologic characteristics of infants with HIE**

| **Variable** | **Frequency (%)** |
| --- | --- |
| **Age of neonate (hours)**  **(median, IQR)** | 26.5 (10 – 53.5) |
| ≤ 24hour | 150 (48.08) |
| 24-48hour | 81 (25.96) |
| ≥72 | 81 (25.96) |
| **Gender** |  |
| Female | 102 (32.7) |
| Male | 210 (67.3) |
| **Gestational age at delivery (weeks)** |  |
| Pre-term (<37) | 14 (4.5) |
| Term (37 - 41) | 296 (94.9) |
| Post-term (>41) | 2(0.6) |
| **Weight (gram)**  **(median, IQR)** | 3000 (2700 – 3475) |
| <2500 | 36 (11.5) |
| 2,500 – 3999 | 258 (82.69) |
| ≥4000 | 18(5.77) |
| **Place of birth** |  |
| Inborn (Booked) | 19 (6.2) |
| Inborn (Unbooked) | 30 (9.8) |
| Outborn | 257 (84.0) |
| Not stated | 6 (1.9) |

**Maternal characteristics**

Information was only available for the inborn mothers with 47 out of the 49 mothers having complete data. The mean maternal age and median parity was 28.02± 5.33 years and 2 (1-3) respectively. Only 6.2% of the mothers booked in LUTH and about four-fifth (n= 38/47, 80.85 %) of the mothers that delivered in LUTH had an emergency caesarean section. (Table 2)

**Table 2. Demographic and obstetrics characteristics of the mothers of inborn infants with HIE**

| **Maternal characteristics** | **Frequency (%)** |
| --- | --- |
| **Booking status of mothers** |  |
| Booked | 19 (6.21) |
| Unbooked | 30 (9.80) |
| Outborn | 257 (83.99) |
| Not stated | 6 (1.92) |
| **Maternal age (years)**  **Mean ± SD** | 28.02± 5.33 |
| <25 | 15(31.91) |
| 25-29 | 8 (17.02) |
| 30-34 | 19 (40.43) |
| 35 – 37 | 5 (10.64) |
| **Parity (median, IQR)** | 2(1-3) |
| 1 | 21 (44.68) |
| 2 | 12 (25.53) |
| 3 | 6 (12.77) |
| 4 | 7 (14.89) |
| 5 | 0(0.00) |
| 6 | 1 (2.13) |
| **Mode of delivery** |  |
| Vaginal delivery | 7(14.89) |
| Assisted Vaginal delivery | 2 (4.26) |
| Emergency Caesarean section | 38 (80.85) |

**Prevalence of HIE, pattern of presentation and outcome**

The prevalence of HIE among newborn admissions during the study period was 7.1% (312/4399). The majority of the infants presented with moderate HIE (n= 190/312, 60.9%, 95%CI:55.34 % – 66.19%), followed by severe HIE (n= 75/312, 24.0% (95%CI:19.60% – 29.12%). Mild cases were the least common (n= 47/312, 15.1% (95%CI: 11.49% - 19.50%) (Fig 1a). Majority (n= 224/312, 71.79%, 95%CI:) of the babies were discharged while the case fatality rate was 25.32% (95%CI: 20.78% – 30.47%, n= 79/312,) (Fig 1b).

**Fig 1a: Pattern of severity of Hypoxic Ischaemic Encephalopathy**

**Fig 1b: Outcome of the Hypoxic Ischaemic Encephalopathy admissions**

**Trends in the annual incidence, mortality, fatality rate and severity of asphyxia (2015 - 2019)**

The number of asphyxia admission decreased from 63 admissions in 2015 to 46 admissions in 2018 and then increased to 86 admissions in 2019. (Figure 2a, S1 Table) Similarly, there was a decrease in the annual asphyxia incidence from 9.17 per 100 neonatal admissions in 2015 to 5.19 per 100 neonatal admissions in 2017 at a rate of 18.4% per annum. Subsequently, there was an increase in incidence from 5.19 per 100 admissions in 2017 to 8.54 per 100 admissions in 2019 at a rate of 74.0% per annum. The overall asphyxia incidence from 2015 (9.17 per 100 neonatal admissions) to 2019 (8.54 per 100 admissions) showed a decline of 1.4% per annum (Figure 2b, S1 Table)

**Fig 2a. Trends in HIE admissions**

**Fig 2b: Trends in incidence and fatality rate for HIE (2015 - 2019)**

The annual fatality rate increased from 17.46% in 2015 to 30.23% in 2019 at a rate of 10.3% per annum. (Figure 2b, S1 Table). Both moderate and severe HIE increased in proportion from 2015 to 2017 then slightly decline in proportion while mild cases decreased from 2015 to 2017 and then slightly increased till 2019. (Figure

**Fig 2c: Trends in severity of admitted cases of HIE (2015 - 2019)**

**Association between severity of HIE and socio biological characteristics**

From Table 3, the median duration of hospital stay was shortest among the babies with severe HIE, 120 (IQR: 24 -240) hours as compared to babies with mild HIE, 240 (IQR: 144 – 240) hours, and moderate HIE, 240 (IQR: 160- 312) hours, respectively (P-value = 0.0002). Furthermore, babies with mild HIE had higher median birth weight (3200 (IQR: 2800 - 3600) g) as compared to babies with moderate 3000 (IQR: 2700 - 3400) or severe HIE 3000 (IQR: 2500 - 3400) g. Although not reaching a statistically significant level. (P-value = 0.075).

**Table 3. Relationship between severity of asphyxia and neonatal and maternal factors**

| **Severity** | **Mild (HIE1)**  **N=47** | **Moderate (HIE2)**  **N=190** | **Severe (HIE3)**  N=75 | **P-value** |
| --- | --- | --- | --- | --- |
| **Age of neonate at presentation(hours)**  **(median, IQR)** | 26(7 - 56) | 34.5(34.5) | 24(8 - 48) | 0.4031 |
| ≤ 24hour | 23 (15.3%) | 85 (57%) | 42 (28%) | 0.336 |
| 24-48hour  (1-2 days) | 11(13.6%) | 52 (64%) | 18 (22.2%) |  |
| 3-7 days | 13 (16.25) | 53 (66.25) | 14 (17.5) |  |
| >14days | 0 (0.0%) | 0 (0.0%) | 1(100) |  |
| **Duration of hospital stay (hour)**  **Median, IQR** | 240 (144 - 240) | 240 (168 - 312) | 120(24 – 240) | 0.0002 |
| **<24 hours** | 2 (4.26) | 22 (11.58) | 23 (30.67) | < 0.001 |
| **24 – 72 hours** | 3 (6.38) | 11 (5.79) | 12 (16.00) |  |
| **>72 hours** | 42 (89.36) | 157 (82.63) | 40 (53.33) |  |
| **Gender** |  |  |  |  |
| Female | 13 (27.66 ) | 68 (35.79) | 21(28.00) | 0.347 |
| Male | 34 (72.34) | 122 (64.21) | 54 (72.00) |  |
| **Gestational age at delivery (weeks)** |  |  |  |  |
| Pre-term (<37) | 4 (8.5) | 6 (3.2) | 4 (5.3) | 0.261 |
| Term (≥37 ) | 43 (91.5) | 184 (96.8) | 71(94.7) |  |
| **Birth weight in gram**  **(median, IQR)** | 3200 (2800 - 3600) | 3000 (2700 - 3400) | 3000 (2500 - 3400) | 0.0756 |
| <2500 | 5 (10.6) | 23 (12.1) | 8 (10.7) | 0.383 |
| 2,500 – 4200 | 38 (80.9) | 161 (84.7) | 66 (88.0) |  |
| ≥ 4200 | 4 (8.5) | 6 (3.2) | 1 (1.3) |  |
| **Maternal age (years)**  **Mean ± SD** | 29.11**±** 4.04 | 28.44 **±** 5.62 | 26.09 **±** 5.49 | 0.3787 |
| <25 | 2 (22.2) | 8 (29.6) | 5 (45.5) | 0.936 |
| 25-29 | 2 (22.2) | 4 (14.8) | 2 (18.2) |  |
| 30-34 | 4 (44.4) | 12 (44.4) | 3 (27.3) |  |
| 35 – 37 | 1 (11.1) | 3 (11.1) | 1 (9.1) |  |
| **Parity (median, IQR)** | 2(2-3) | 2(1-3) | 1(1-2) | 0.0983 |
| 1 | 2 (22.2) | 11 (40.7) | 8 (72.7) | 0.424 |
| 2 | 4 (36.4) | 6 (22.2) | 2 (18.8) |  |
| 3 | 1 (11.1) | 5 (18.5) | 0 (0.0) |  |
| 4 | 2 (22.2) | 4 (14.8) | 1 (9.1) |  |
| 5 | 0 (0.0) | 0 (0.0) | 0 (0.0) |  |
| 6 | 0 (0.0) | 1 (3.5) | 0 (0.0) |  |
| **Mode of delivery** |  |  |  |  |
| Vaginal delivery | 1 (11.1) | 6 (22.2) | 0 (0.0) | 0.397 |
| Assisted Vaginal delivery | 0 (0.0) | 1 (3.7) | 1 () |  |
| Emergency Caesarean section | 8 (88.9) | 20 (74.1) | 10 () |  |
| **Place of birth** |  |  |  |  |
| Inborn (Booked) | 5 (11.6) | 13 (6.8) | 1 (1.4) | 0.075 |
| Inborn (Unbooked) | 5 (11.6) | 14 (7.4) | 11 (14.9) |  |
| Outborn | 33 (76.7) | 162 (85.7) | 62 (83.8) |  |
| **Categories of place of birth** |  |  |  |  |
| Booked (LUTH) | 5 (10.6) | 13 (6.8) | 1 (1.3) | 0.001 |
| Unbooked (LUTH) | 5 (10.6) | 14 (7.3) | 11 (14.7) |  |
| Public Secondary Health facility | 3 (6.4) | 8 (4.2) | 2 (2.7) |  |
| Private Health facility | 20 (42.5) | 80 (42.1) | 30 (40) |  |
| Maternity Home | 4 (8.5) | 32 (16.8) | 14 (18.7) |  |
| Primary Health Centre | 5 (10.6) | 22 (11.6) | 2 (2.7) |  |
| TBA/Home | 1 (2.1) | 20 (10.5) | 14 (18.7) |  |
| Not stated | 4 (8.5) | 1 (0.5) | 1 (1.3) |  |

**Association between survival of asphyxiated babies and maternal/neonatal characteristics**

The prevalence of death increased with increasing severity of HIE and the prevalence of death was about three to four fold among babies with severe cases (53.3%, (95%CI: 41.8% - 64.5%, n=40/75) as compared to babies with mild (12.8% (95%CI: 5.7% - 26.3%, n=6/47), or moderate HIE cases (17.4% (95%CI: 12.6% - 23.5%, n=33/190) (P-value < 0.0001). Babies that survived had longer hospital stay than those that died. 24 (24 - 28) hours Vs 240(192 - 336) hours, P-value < 0.001. Out of the 47 babies with mild HIE, 6 (12.8%) died, while 33 out of the 190 (17.4%) and 40 out of the 75(53.3%) babies with moderate and severe HIE respectively, died. (Table 4)

**Table 4. Association between survival of asphyxiated babies and maternal/neonatal characteristics**

| **Severity** | **Dead** | **Survived/Discharged** | **P-value^a^** |
| --- | --- | --- | --- |
| **Duration of hospital stay (hour)**  **Median, IQR** | 24 (24 - 28) | 240(192 - 336) | <0.0001^b^ |
| **<24 hours** | 44 (55.7) | 3 (1.3) |  |
| **24 – 72 hours** | 23 (29.1) | 3 (1.3) |  |
| **>72 hours** | 12 (15.2) | 227 (97.4) |  |
| Mild (HE1) | 6(17.60) | 41(7.59) | <0.0001 |
| Moderate (HIE2) | 33 (67.38) | 157 (41.77) |  |
| Severe (HE3) | 40 (15.02) | 35(50.63) |  |
| **Age of neonate at presentation(hours)**  **(median, IQR)** | 22(6 -48) | 36 (13 - 72) | 0.0052 ^b^ |
| ≤ 24hour | 49 (62.0) | 101(43.3) | 0.038 |
| 24-48hour  (1-2 days) | 15 (19.0) | 66 (28.3) |  |
| 3-7 days | 15 (19.0) | 65 (28.0) |  |
| >14days | 0 (0.0) | 1 (0.4) |  |
| **Gender** |  |  |  |
| Female | 26 (33.0) | 76 (32.6) | 0.962 |
| Male | 53 (67.1) | 157 (67.4) |  |
| **Gestational age at delivery** |  |  |  |
| Preterm | 7 (8.9) | 7 (3.0) | 0.030 |
| Term | 72 (91.1) | 226 (97.0) |  |
| **Weight (gram)**  **(median, IQR)** | 3000 (2500 - 3500) | 3000 (2800 - 3450) | 0.1730 |
| <2500 | 10 (12.7) | 26 (11.2) | 0.634 |
| 2,500 – 4200 | 65 (82.3) | 200 (85.8) |  |
| ≥ 4200 | 4 (5.1) | 7 (3.0) |  |
| **Maternal age (years)**  **Mean ± SD** | 26.07 **±** 5.48 | 28.94 **±** 5.09 | 0.0855^c^ |
| <25 | 7 (46.7) | 8 (25.0) | 0.395 |
| 25-29 | 3 (20.0) | 5 (15.6) |  |
| 30-34 | 4 (26.7) | 15 (46.9) |  |
| 35 – 37 | 1 (6.7) | 4 (11.8) |  |
| **Parity (median, IQR)** | 1(1-2) | 2 (1-3) | 0.0114 ^b^ |
| 1 | 11(73.3) | 10 (30.3) | 0.128 |
| 2 | 2 (13.3) | 10 (30.3) |  |
| 3 | 1 (6.7) | 5 (15.2) |  |
| 4 | 1 (6.7) | 6 (18.2) |  |
| 5 | 0 (0.0) | 0 (0.0) |  |
| 6 | 0 (0.0) | 1 (3.0) |  |
| **Mode of delivery** |  |  |  |
| Vaginal delivery | 1 (6.7) | 6 (18.8) | 0.358 |
| Assisted Vaginal delivery | 0 (0.0) | 2 (6.3) |  |
| Emergency Caesarean section | 14 (93.3) | 24 (75.0) |  |
| **Place of birth** |  |  |  |
| Inborn (booked) | 1 (1.3) | 18 (7.9) | 0.001 |
| Inborn (Unbooked) | 15 (19.2) | 15 (6.6) |  |
| Outborn | 62 (79.5) | 195 (85.5) |  |
| **Facilities of birth** |  |  |  |
| Booked (LUTH) | 1 (1.3) | 18 (7.9) | < 0.0001 |
| Unbooked (LUTH) | 15 (19.2) | 15 (6.6) |  |
| Public Secondary Health facility | 0 (0.0) | 13 (5.7) |  |
| Private Health facility | 26 (33.3) | 104 (45.6) |  |
| Maternity Home | 7 (9.0) | 43 (18.9) |  |
| Primary Health Centre | 9 (11.5) | 20 (8.8) |  |
| TBA/Home | 20(57%) | 15(43%) |  |
| **Year of admission** |  |  |  |
| 2015 | 11(13.92) | 52 (22.32) | 0.519 |
| 2016 | 18 (22.8) | 50 (21.46) |  |
| 2017 | 12 (15.2) | 37 (15.9) |  |
| 2018 | 12 (15.2) | 34 (14.6) |  |
| 2019 | 26 (33.0) | 60 (25.8) |  |
| ^a^Except otherwise stated, logrank test. ^b^ Mann Whitney U test, ^c^Student’s t-test | | | |

**Survival experience of the babies with HIE**

The overall survival experience of the cohort is as shown in Figure 3(A). The babies contributed 2745.96 neonate-days of follow-up and the median time to death was 24(24 – 48) days. The death rate was 28.8 per 1000 neonate-days. About 14.1% (n= 44/312) died within the first 24 hours of admission while 21.5% (n= 67/312) died within 72 hours. About 75.0% of the babies survived beyond 10 days. The cumulative risk of survival at 3rd,7th and 14th day of admission was 85.8%, (95%CI:81.4% - 89.2%), 78.4% (95% CI: 73.3% - 82.5%) and 74.7%, (95%CI: 69.4% - 79.3%) respectively. (S2 table)

**Fig 3. The Kaplan-Meier survival experience of the cohort (A), severity of HIE (B), by place of birth (C), and year of delivery (D)**

Figure 3(B) showed the survival experience of the babies based on severity of disease. Babies with severe disease died faster than the others with 75% and 50% of severe cases respectively surviving beyond the 1st and 7^th^ day of admission. While 75% of moderate cases survived beyond 24 days. The mortality rate by severity was 14.0 per 1000 neonate-days, 18.0 per 1000 neonate-days and 82.0 per 1000 neonate-days for mild, moderate and severe cases respectively. (Log-rank p-value < 0.001).

Based on the Kaplan-Meier survival estimates on the place of birth, babies who were classified as unbooked inborn deliveries had the worst survival experience while the booked inborn had lowest deaths. Thus, the mortality rate was 7.0 per 1000 neonate-days, 110.1 per 1000 neonate-days and 25.7 per 1000 neonate-days for booked inborn, unbooked inborn and outborn babies respectively. About 50% of the unbooked inborn babies survived beyond 2 days of admission while 50% of the outborn survived beyond 24 days. About 94.5% of the booked inborn survived and were discharged. (Fig 3C). There was no statistically significant difference in the survival by year of admission. (Fig 3D)

**COX proportional hazard of death among babies admitted with HIE**

On univariable Cox proportional regression, babies who were classified as booked inborn had about 91% lesser hazard of death from asphyxia as compared to babies classified as unbooked inborn (HR 0.09, 95%CI:0.01 - 0.66, P-value = 0.02). Furthermore, babies that were delivered preterm had about 2.8-fold hazard of death from asphyxia as compared to admitted term babies (HR 2.79, 95%CI: 1.28- 6.07, P-value = 0.01). The hazard of death among asphyxiated babies who were aged 24 – 48 years at presentation were about 50% less likely as compared to babies whose age at presentation was less than 24 hours. (HR 0.50, 95%CI:0.28 - 0.90, P-value = 0.021). When taking age as a continuous variable, we found that for every hour increase in age at presentation, the hazard of death decreases by 1% (HR 0.99, 95%CI: 0.979 - 0.996, P-value = 0.005). The hazard of death was 91% lesser among babies who were “booked inborn” (HR 0.09, 95%CI: 0.01 - 0.66, P-value = 0.018) as compared to babies of unbooked inborn. Furthermore, outborn babies had 62% lesser hazards of deaths as compared to inborn unbooked babies (HR 0.38, 95%CI: 0.21 - 0.66, P-value = 0.001). For every unit increase in the parity of the mothers of the babies, the hazard of death among the asphyxiate babies decreases by 49% (HR 0.51, 95%CI: 0.270-0.997, P-value = 0.049). Other statistically significant relationship after univariable regression includes gestational age at delivery. Babies’s gender, maternal age and mode of delivery did not influence the hazard of death among the babies (Table 5)

After multivariable Cox proportional hazard regression and adjusting for severity, gender, weight. Age at presentation, type of facility, year of admission (Table 5), we found that the hazard of death among babies with severe HIE (HIE3) was about 4 times the hazard of death among infants with mild HIE (adjHR 4.17,95%CI: 1.72 - 10.11, P-value = 0.002). However, there was no statistically significant difference in the hazard of death among infants with moderate asphyxia as compared with babies with mild asphyxia. Furthermore, children who were unbooked inborn had about 9-fold hazard of death as compared to booked inborn children (adjHR 13.65,95%CI: 1.74 - 107.32, P-value = 0.01) while babies who were delivered at TBA or home had about 14-fold hazard of death as compared to babies delivered by booked women in LUTH (adjHR 8.98,95%CI: 1.15 - 69.85, P-value = 0.036). The hazard of death increased with increasing year of admission but slightly declined in 2019.

**Table 5. Univariate and multivariable regression analysis of predictors of outcome among the HIE infants**

| **Variable** | **Univariable** | | | **^a^Multivariable** | | |
| --- | --- | --- | --- | --- | --- | --- |
|  | **HR** | **95%CI** | **p-value** | **AdjHR** | **95%CI** | **p-value** |
| **Severity of Asphyxia** | | | | | | |
| Mild (HIE1) | 1.00 | Ref | Ref | 1.00 | Ref | Ref |
| Moderate (HIE2) | 1.38 | 0.58 - 3.29 | 0.471 | 1.12 | 0.46 - 2.76 | 0.797 |
| Severe (HE3) | 5.08 | 2.15 - 12.00 | < 0.001 | 4.17 | 1.72 - 10.11 | 0.002 |
| **Age of neonate at presentation** | 0.99 | 0.979 - 0.996 | 0.005 |  |  |  |
| ≤ 24hour | 1.00 | Ref | Ref | 1.00 | Ref | Ref |
| 24-48hour | 0.50 | 0.28 - 0.90 | 0.021 | 0.55 | 0.29 - 1.04 | 0.068 |
| >48 hours | 0.49 | 0.27 - 0.87 | 0.016 | 0.75 | 0.39 - 1.43 | 0.382 |
| **Gender** |  |  |  |  |  |  |
| Male | 1.00 | Ref | Ref | 1.00 | Ref | Ref |
| Female | 1.03 | 0.64 - 1.64 | 0.916 | 1.16 | 0.70 1.94 | 0.564 |
| **Weight (gram)**  **(median, IQR)** | 0.9997 | 0.999-1.000 | 0.196 |  |  |  |
| ≥2,500 | 1.00 | Ref | Ref | 1.00 | Ref | Ref |
| <2500 | 1.23 | 0.63 - 2.39 | 0.540 | 1.04 | 0.51-2.09 | 0.923 |
| **Facilities of birth** | . |  |  |  |  |  |
| Booked (LUTH) | 1.00 | Ref | Ref | 1.00 | Ref | Ref |
| Unbooked (LUTH) | 11.41 | 1.50 - 86.66 | 0.019 | 8.98 | 1.15 - 69.85 | 0.036 |
| Public Secondary / primary Health facility | 3.91 | 0.50 - 30.88 | 0.196 | 4.09 | 0.49 - 33.80 | 0.191 |
| Private Health facility | 3.39 | 0.46 - 25.03 | 0.230 | 3.02 | 0.39 - 23.15 | 0.287 |
| Maternity Home | 2.33 | 0.29 - 18.98 | 0.428 | 2.00 | 0.23 - 17.14 | 0.526 |
| TBA/Home | 13.19 | 1.77 - 98.37 | 0.012 | 13.65 | 1.74 - 107.32 | 0.013 |
| **Year of admission** |  |  |  |  |  |  |
| 2015 | 1.00 | Ref | Ref | 1.00 | Ref | Ref |
| 2016 | 1.49 | 0.70 - 3.16 | 0.297 | 1.93 | 0.88 - 4.24 | 0.100 |
| 2017 | 1.38 | 0.60 - 3.16 | 0.442 | 1.94 | 0.80 - 4.72 | 0.145 |
| 2018 | 1.51 | 0.67 - 3.43 | 0.321 | 2.78 | 1.19 - 6.53 | 0.019 |
| 2019 | 1.72 | 0.85- 3.49 | 0.130 | 2.66 | 1.23 - 5.75 | 0.013 |
| **Gestational age at delivery (weeks)** |  |  |  |  |  |  |
| Term (≥37) | 1.00 | Ref | Ref |  |  |  |
| Pre-term | 2.79 | 1.28- 6.07 | 0.010 |  |  |  |
| **Place of birth** |  |  |  |  |  |  |
| Inborn (Unbooked) | 1.00 | Ref | Ref |  |  |  |
| Inborn (booked) | 0.09 | 0.01 - 0.66 | 0.018 |  |  |  |
| Outborn | 0.38 | 0.21 - 0.66 | 0.001 |  |  |  |
| **Maternal age (years)** | 0.93 | 0.85 - 1.02 | 0.126 |  |  |  |
| <25 | 1.00 | Ref | Ref |  |  |  |
| 25-29 | 0.71 | 0.18 - 2.75 | 0.621 |  |  |  |
| 30-34 | 0.40 | 0.12 - 1.38 | 0.147 |  |  |  |
| 35 – 37 | 0.42 | 0.05 - 3.39 | 0.413 |  |  |  |
| **Parity** | 0.51 | 0.270 0.997 | 0.049 |  |  |  |
| 1 | 1.00 | Ref | Ref |  |  |  |
| 2 | 0.25 | 0.05 - 1.11 | 0.068 |  |  |  |
| 3 | 0.30 | 0.04 - 2.31 | 0.247 |  |  |  |
| 4 | 0.24 | 0.03 - 1.87 | 0.174 |  |  |  |
| 6 | 2.73 x 10^-16^ | 0 | 1.000 |  |  |  |
| **Mode of delivery** |  |  |  |  |  |  |
| Vaginal delivery | 1.00 | Ref | Ref |  |  |  |
| Emergency Caesarean section | 3.44 | 0.45 - 26.17 | 0.233 |  |  |  |
| **Place of birth** |  |  |  |  |  |  |
| Inborn (Unbooked) | 1.00 | Ref | Ref |  |  |  |
| Inborn (booked) | 0.09 | 0.01 - 0.66 | 0.018 |  |  |  |
| Outborn | 0.38 | 0.21 - 0.66 | 0.001 |  |  |  |
| HR - Hazard ratio; AdjHR – Adjusted hazard ratio; CI – Confidence interval; TBA - Traditional birth attendant. **^a^**Model adjusted for severity, gender, weight. Age at presentation, type of facility, year of admission | | | | | | |

The Schoenfeld’s proportional test assumptions gave a p-value of 0.5153 which showed that there was no violation of the assumptions of Cox proportional hazard. The variance inflation factor was 1.03 showing that there was no collinearity among the variables.

**Discussion**

This study assessed the temporal trend in perinatal asphyxia and some of the predictors of immediate in-hospital mortality among newborns with HIE admitted into the neonatal units of a tertiary hospital in Lagos, Nigeria, over five years. We observed an initial decline in annual asphyxia incidence from 2015 to 2017, before it increased from 2018 to 2019. Place of delivery, booking status of the mother, gestational age at birth, age of infant at presentation and severity of HIE were all found to significantly predict in-hospital mortality.

There is national and regional variation in the prevalence and fatality rate of HIE in literature, from less than 1% in developed countries to as high as 25% in some poor countries, possibly due to the varying quality of maternity care. [ 2,18-21] The prevalence of HIE was 7.1% among newborn admissions in the present study with a case fatality rate of 25.3%. This is higher than the prevalence reported in most developed countries but similar to what poorer African nations reported. [3,19-22,27,28] Though the prevalence was also higher than the 3.3% reported by Ugwu et al [5] in Niger Delta, Nigeria, it is lower than the 21.1% reported by Ila et al [21] in Zamfara state, and Ekwochi et al [22] in Enugu state, both in Nigeria. These disparities may be related to the quality of obstetric and neonatal care available in these places. [18-23] On the other hand, the case fatality rate of 25.3% among our cohort of babies was similar to that reported by Ugwu et al [5] and Ila et al [21] but higher than that reported by Ogunkunle et al, [24 all in Nigeria. The higher case fatality rate recorded in our study may be due to our inclusion criteria. Only babies with HIE were included in our study unlike other similar studies in Nigeria that recruited infants based on APGAR scoring system. [5,10,16-18] The use of APGAR scores to determine asphyxia is marred by several well-documented limitations. [25,26] In affluent societies with good facilities for impeccable neonatal care, HIE and its associated morbidities and mortalities have drastically reduced such that the case fatality rate of HIE was minimal [23,27,28]

Appropriate training in neonatal resuscitation can reduce neonatal mortality thereby considerably reducing the under-five mortality trends, especially in resource-poor countries such as Nigeria. [29] In Nigeria, activities around newborn resuscitation training and neonatal care increased markedly from 2010. [14,15] In Lagos, the years 2016-2018 marked the peak of neonatal resuscitation and “helping babies breathe” training with nearly all the health facilities in Lagos (both private and publicly funded health facilities) benefiting from the series of trainings. Our study showed a reduction in the incidence per 100 admissions of asphyxiated infants during the same periods of the neonatal resuscitation training in Lagos (2015 – 2017). Since more than 80% of babies admitted for asphyxia at our centre were referred from other health facilities, the observed reduction may suggest a population or community level decline in HIE for the period. There was, however, a surge in the incidence of HIE from 2018 to 2019. This later upward trend calls for concerted efforts to arrest the trend to reduce morbidity and mortality from asphyxia. This trend will also set back all the gains achieved in reducing neonatal mortality rate in Nigeria and further derail the sustainable Development Goals (SDG) 3.2. Our result highlights the need for Nigeria to strive towards reducing child mortality through a reduction in neonatal mortality. [29,30] Furthermore, our study suggests that training and retraining of healthcare providers on newborn resuscitation skills is necessary to reduce newborn mortality. Stakeholders should make provisions in the training guidelines of healthcare workers that requires newborn care providers to repeat neonatal resuscitation training at intervals. The neonatal resuscitation guidelines of the American Academy of Pediatrics strongly advise a two-yearly certification and refresher trainings on neonatal resuscitation skills for providers. [32] Nigeria may borrow a leaf from the American guideline, as it will ensure a workforce that is confident and adequately equipped to care for these vulnerable infants at birth.

Since the neonatal period is the most vulnerable time for a child’s survival, [29] our study highlighted the vulnerability of the first day of life and the high risk for mortality it imposed on asphyxiated newborns. Our study reported 14% mortality within the first 24hours of life and this was coroborated by other studies that reported similar trends. [10,24,32] As expected, the worse the survival of the babies was inversely related to the HIE severity. Our study noted that the hazard of death in infants that suffered severe HIE was about 4 times the hazard of death among infants with mild asphyxia. It is pertinent to note that even mild forms of HIE can progress to mortality. This is important because many interventions for HIE tend to exclude HIE stage 1. [33,34] For example, the international guidelines for therapeutic hypothermia for facilities with the resources for this treatment excludes infants with HIE 1. Though management of HIE in our center is still supportive, [13] in developed countries that undertake body cooling for HIE, cooling is usually instituted for HIE 2 and 3 only. The implication is that these infants with initial mild diseases miss out on treatment opportunities because they did not meet the treatment criteria within the first 6 hours of life. However, with the progression of secondary energy failure which can last up to 72 hours these mild diseases may deteriorate to severe HIE and ultimate demise. There is a need to constantly monitor these infants and possibly institute other adjuvant treatments such as Erythropoietin for them. This is even more pertinent in developing countries like Nigeria where more than 60% of births take place in the home or under the care of unskilled birth attendants [14,15] and the asphyxiated infants present late to the hospital. [30] The present study showed that majority of the infants with HIE were outborn and that most of the babies delivered at home or in a traditional birth attendant’s place, who presented with HIE, died. In the same vain peripartum referral of a high-risk pregnancy at the point of delivery is fraught with dangers. Though many of the outcome of such pregnancies come out favourable after expert management but a great majority suffer adverse events. Miyoshi in Zambia noted that cesarean sections performed emergently in referred mothers led to poorer perinatal outcomes in the infants. [35]

Our study noted that the survival experience of babies of unbooked mothers in our facility is very poor with only half of the severely asphyxiated neonates from such deliveries surviving beyond two days. This is similar to other studies in Nigeria and elsewhere that documented this phenomenon. [6,22,35,36] We further documented a 9-fold hazard of death among infants who were unbooked inborn as compared to booked inborn children. Conversely, inborn neonates of booked mothers had about 91% lesser hazard of death from asphyxia. This is significant and buttresses the point that high-risk pregnancies should be identified early and referred to facilities where the mother can be adequately managed and stabilized long before delivery. This may also reduce the incidence of emergency cesarean sections and improve neonatal outcome. Furthermore, delivery at home or in a TBA confers a 14-fold hazard of death on such babies as compared to inborn babies delivered by booked women among our cohort of asphyxiated babies.

Our study did not examine the co-morbidities of the babies while on admission. Other morbidities may contribute to the recorded mortalities. Another limitation of this study was its retrospective nature, and, as such it has all the challenges such as the inability to retrieve all demographic data for outborn mothers. However, despite these limitations, this study was the first to study the trends in perinatal asphyxia incidence in Nigeria and linked it with neonatal resuscitation training while demonstrating the need for regular and recurrent training of providers of newborn care. Our study focused only on infants with clinical evidence of acute neurological dysfunction or complications of perinatal asphyxia diagnosed using standard instruments for HIE, thereby bypassing the pitfalls inherent in diagnosing perinatal asphyxia with APGAR scoring.

Overall, our study findings suggest that sustaining newborn resuscitation skills, as well as proper management of perinatal complications, are critical for saving newborn lives and reducing neonatal mortality in Nigeria.

**Conclusion**

HIE is prevalent as a cause of morbidity and mortality in Nigerian newborns with waning and waxing trends. Some maternal and neonatal factors, as well as the severity of the HIE, were found to significantly predict in-hospital mortality. There is need to sustain the training and retraining of healthcare providers on newborn resuscitation skills and develop curriculum guidelines that require retraining on neonatal resuscitation at intervals of two years for newborn care providers in Nigeria as obtainable elsewhere.

**Acknowledgement:** GO is funded by the Glaxo Smith Kline/ Sub-Saharan Africa Consortium for Advanced Biostatistics training/DELTAS Africa Fellowship through the School of Public Health, University of Witwatersrand. The views expressed are those of the authors and not necessarily that of the funders.

**References**

1. Lawn JE, Manandhar A, Haws RA, Darmstadt GL. Reducing one million child deaths from birth asphyxia: a survey of health systems gaps and priorities. Health Res Policy Syst. 2007; 5:4.
2. Aliyu I, Lawal TO, Onankpa B. Prevalence and outcome of perinatal asphyxia: Our experience in a semi –urban setting. Tropical journal of Medical Research. 2017;20(2):161-165.
3. Alemu A, Melaku G, Abera G.B, Damte A. Prevalence and associated factors of perinatal asphyxia among newborns In Dilla University referral hospital, Southern Ethopia-2017.2019;10:69-74.
4. Ogunlesi TA, Fetuga MB, Adekanmbi AF. Mother knowledge about birth asphyxia: The need to do more. Niger J Clin Pract.2013;16:31-6.
5. Mcgil Ugwu GI, Abedi HO, Ugwu EN. Incidence of Birth Asphyxia as seen in Central Hospital and GN Children’s Clinic both in Warri Niger Delta of Nigeria: An Eight Year Retrospective Review. Glob J Health Sci.2012;4(5):140-146.
6. Onyearugha C.N, Ugboma H.A.A. Severe birth asphyxia: risk factors as seen in a tertiary institution in the Niger Delta area of Nigeria. Continental J. Tropical Medicine.2010;4:11-19.
7. Zulfiqar A. Bhutta. Birth Asphyxia. Manson’s Tropical Infectious Disease.2014.
8. Gillam-Krakauer M, Gowen Jr CW. Birth Asphyxia. [Updated 2020 Apr 23]. In: StatPearls [Internet]. Treasure Island (FL): StatPearls Publishing; 2020. Available from: https://www.ncbi.nlm.nih.gov/books/NBK430782/
9. Egharevba OI, Kayode-Adedeji BO, Alikah SO. Perinatal asphyxia in a rural Nigerian hospital: Incidence and determinants of early outcome. J Neonatal Perinatal Med.2018;11(2):179-183.
10. Uleanya ND, Aniwada EC, Ekwochi U, Uleanya ND. Short term outcome and predictors of survival among birth asphyxiated babies at a tertiary academic hospital in Enugu, South East, Nigeria. Afr Health Sci. 2019;19(1):1554-1562. doi:10.4314/ahs.v19i1.29
11. WHO. Newborns: reducing mortality WHO 2019. <https://www.who.int/news-room/fact-sheets/detail/newborns-reducing-mortality>
12. Sarnat HB, Sarnat MS. Neonatal encephalopathy following fetal distress. A clinical and electroencephalographic study. Arch. Neurol. 1976 Oct.;33(10):696–705.
13. Ezenwa B, Ezeaka C, Fajolu I, Ogbenna A, Olowoyeye O, Nwaiwu O, et al. Impact of Erythropoietin in the management of Hypoxic Ischaemic Encephalopathy in resource-constrained settings: protocol for a randomized control trial. BMC Neurology. 2020 May 4; 20: 171
14. Federal Ministry of Health. Nigeria Every Newborn Action Plan: A plan to end preventable newborn deaths in Nigeria. Abuja: Federal Ministry of Health; 2016.
15. Federal Ministry of Health. Saving newborn lives in Nigeria: Newborn health in the context of the Integrated Maternal, Newborn and Child Health Strategy. 2nd edition. Abuja: Federal Ministry of Health, Save the Children, Jhpiego; 2011.
16. Aluko JO, Modeste RR, Adejumo O, Anthea R. Return for prenatal care and childbirth services among Nigerian women using primary health care facilities. Nurs Open. 2019;7(1):91-99. doi:10.1002/nop2.314
17. World Population review. Lagos Population 2020 (Demographics, Maps, Graphs) <https://worldpopulationreview.com/world-cities/lagos-population>
18. Arnaez J, García-Alix A, Arca G, Valverde E, Caserío S, Moral MT, et al. Incidencia de la encefalopatía hipóxico-isquémica e implementación de la hipotermia terapéutica por regiones en Espana. ˜ An Pediatr (Barc). 2018;89:12---23.
19. Namusoke H, Nannyonga MM, Ssebunya R, Nakibuuka VK, Mworozi E. Incidence and short term outcomes of neonates with hypoxic ischemic encephalopathy in a Peri Urban teaching hospital, Uganda: a prospective cohort study. Matern Health Neonatol Perinatol. 2018;4:6. Published 2018 Mar 7. doi:10.1186/s40748-018-0074-4
20. Simiyu IN, Mchaile DN, Katsongeri K, Philemon RN, Msuya SE. Prevalence, severity and early outcomes of hypoxic ischemic encephalopathy among newborns at a tertiary hospital, in northern Tanzania. BMC Pediatr. 2017;17(1):131. Published 2017 May 25. doi:10.1186/s12887-017-0876-y
21. Ilah BG, Aminu MS, Musa A, Adelakun MB, Adeniji AO, Kolawole T. Prevalence and Risk Factors for Perinatal Asphyxia as Seen at a Specialist Hospital in Gusau, Nigeria. Sub-Saharan Afr J Med 2015; 2:64-9.
22. Ekwochi U, Asinobi NI, Osuorah CD, Ndu IK, Ifediora C, Amadi OF, et al. Incidence and Predictors of Mortality Among Newborns with Perinatal Asphyxia: A 4-Year Prospective Study of Newborns Delivered in Health Care Facilities in Enugu, South-East Nigeria. Clinical Medicine Insights: Pediatrics. 2017; 11: 1–10
23. Cruz AC, Ceccon ME. Prevalence of asphyxia and perinatal hypoxic-ischemic encephalopathy in term newborns, considering two diagnostic criteria. Rev. bras. crescimento desenvolv. hum. [online]. 2010; 20(20: 302-316.
24. Ogunkunle TO, Odiachi H, Chuma JR, Bello SO, Imam A. Postnatal Outcomes and Risk Factors for In-Hospital Mortality among Asphyxiated Newborns in a Low-Resource Hospital Setting: Experience from North-Central Nigeria. Annals of Global Health. 2020; 86(1): 63, 1–9. DOI: https://doi.org/10.5334/aogh.2884
25. Jain L. Severe Perinatal Asphyxia and Apgar Scores. Am J Dis Child. 1987;141(12):1253. doi:10.1001/archpedi.1987.04460120015013
26. American Academy of Pediatrics, Committee on Fetus and Newborn; American College of Obstetricians and Gynecologists and Committee on Obstetric Practice. The Apgar score. Pediatrics. 2015, 136 (4) 819-822; DOI: https://doi.org/10.1542/peds.2015-2651
27. Smith J, Wells L, Dodd K. The continuing fall in incidence of hypoxic-ischaemic encephalopathy in term infants. BJOG. 2000;107(4):461-466. doi:10.1111/j.1471-0528.2000.tb13262.
28. Ouwehand S, Smidt LCA, Dudink J, et al. Predictors of Outcomes in Hypoxic-Ischemic Encephalopathy following Hypothermia: A Meta-Analysis [published online ahead of print, 2020 Apr 1]. Neonatology. 2020;1-17. doi:10.1159/000505519
29. Hug L, Lee S, Sharrow D, You D. Levels & Trends in Child Mortality Report 2019. Estimates Developed by the UN Inter-Agency Group for Child Mortality Estimation. 2019. https://www.unicef. org/media/60561/file/UN-IGME-child-mortalityreport-2019.pdf. Accessed July 11, 2020.
30. National Population Commission (NPC) [Nigeria] and ICF. 2019. Nigeria Demographic and Health Survey 2018. Abuja, Nigeria, and Rockville, Maryland, USA: NPC and ICF.
31. Weiner GM, Zaichkin J, editors. Textbook of neonatal resuscitation (NRP), 7^th^ Ed. American Academy of Pediatrics and American Heart Association. 2016
32. Lawn, Joy E., et al., ‘Every Newborn: Progress, priorities, and potential beyond survival’, The Lancet, vol. 384, no. 9938, 12 July 2014, pp. 189-205.
33. Lemyre B, Chau V, Canadian Paediatric Society, [Fetus and Newborn Committee](https://www.cps.ca/en/documents/authors-auteurs/fetus-and-newborn-committee). Hypothermia for newborns with hypoxic-ischemic encephalopathy. Paediatr Child Health. 2018; 23(4):285–291.
34. Mosalli R. Whole body cooling for infants with hypoxic-ischemic encephalopathy. J Clin Neonatol. 2012;1(2):101-106. doi:10.4103/2249-4847.96777
35. Miyoshi Y. Maternal and perinatal outcomes in urgent referral and non-referral cases of emergency cesarean section at a district hospital in Zambia: a retrospective observational study. Pan African Medical Journal. 2019; 33:324. doi:10.11604/pamj.2019.33.324.19832
36. Dalal CA, Bodar NL. A study on birth asphyxia at tertiary health centre. Natl J Med Res 2013; 3:374-6.

**Supporting information**

**S1 Table. Trends in admission, asphyxia and fatality**

**S2 Table. Life table of the survival experience of the babies with HIE**
