# Supplementary material for: Trends and predictors of in-hospital mortality among babies with hypoxic ischaemic encephalopathy at a tertiary hospital in Nigeria: A retrospective cohort study
Source: PLoS One. 2021 Apr 26;16(4):e0250633. doi: 10.1371/journal.pone.0250633 (PMC8075215; doi:10.1371/journal.pone.0250633)
Supplement: Attachment — Submitted filename: Response to Reviewers.docx [file pone.0250633.s005.docx]

Department of Paediatrics,

College of Medicine,

University of Lagos

21^st^ March, 2021

Dear Editor,

PLoS One.

Dear Sir,

**Response to reviewers’ comments on the manuscript titled ‘Trends and predictors of in-hospital mortality among babies with hypoxic ischaemic encephalopathy at a tertiary hospital in Nigeria: a retrospective cohort study’**

Once more, we are grateful for considering the above titled manuscript for possible publication in your prestigious journal. We have addressed all the further comments of the reviewers as outlined below:

**Reviewers’ comments**

**Reviewer #2:**

As stated for my initial review, this is an important hospital-based study that attempts to address important and complex factors influencing neonatal morbidity (and maternal mortalities!), still more profoundly expressed in resource-poor nations! Thank-you to the authors for incorporating Reviewer 2's suggestions. Other than proofing the manuscript for occasional word corrections in tense, etc in several instances (e.g. line 48,) , this revised manuscript is acceptable for publication.

The challenges for my colleagues who present their research findings regarding maternal- child care before and after birth need constant scrutiny and attention through peer-reviewed dialogue to achieve world-wide medical equity. These challenges existed during the latter part of the 20th century following WWII and were addressed by nations with adequate research and public health resources to reduce mortalities and improve morbidities in their nations (e.g. Collaborative Perinatal Project in the US). Despite obvious successes based on these birth cohort efforts, new challenges have either replaced or sustained the former ones given lack of resources even within these nations. Partnerships among healthcare, social-support agencies and government are needed to merge priorities that will ultimately improve both medical and socioeconomic health for an entire nation's citizenry.
Good luck for future contributions.

Authors’ response: Thank you for your comments, insights and encouragement

**Reviewer #3:**

**Reviewer #3: General comments**
This is a hospital-based study largely of a subpopulation of referred infants with HIE in a large city where there are other hospitals receiving referred infants. The observed trends and changes over time could be due to referral bias. This is now addressed well in the Discussion but not in the Abstract (especially in the Conclusions).

Authors’ response: Thank you for your comments. The conclusion of the abstract has been revised to include the fact that majority of our HIE babies were outborn which may in turn impact on the pattern of the burden of the HIE at our centre. Thus, the conclusion of the abstract now states “. The case fatality rate of HIE is high and increasing at our centre and mainly driven by the pattern of admission of HIE cases among outborn babies. Thus, community level interventions including skilled birth attendants at delivery, newborn resuscitation trainings for healthcare personnel and capacity building for specialized care should be intensified to reduce the burden of HIE from perinatal asphyxia”.

**Reviewer #3 comment:** Abstract
The abstract needs to address the potential for referral bias.
This is a hospital-based study conducted retrospectively and covering many years. The authors must be more careful when addressing the incidence and fatality rate of asphyxia and refer to hospital admissions rather than make general unqualified statements such as “The annual incidence declined by 1.4% per annum while the annual fatality rate increased by 10.3% per annum from 2015 to 2019.”

Authors’ response: The abstract has been revised and changes were made in line with the suggestion of the reviewer. The objective of the study was made more concise to reflect the data was collected from only one hospital.
In the results section we added phrases such as “ at our centre”, “among the hospital admissions” to reflect that our results and conclusions were based on data obtained from our facility.

**Reviewer #3 comment:** About four fifths of the admissions with HIE were outborn babies. Their mortality rate was four-fold that of inborn babies. The referral bias for mortality if also extremely important.

Authors’ response: Thank you for the kind suggestion. We have incorporated this idea into the conclusion section.

**Reviewer #3 comment:** Introduction
The Introduction is based on rather low level of evidence studies and reviews rather than strong data on HIE from well designed RCTs, cohort studies, and meta-analyses. This was commented in the previous review but not addressed.

There are major studies from multi-country (LMICs) populations as well as some single country (LMIC) that should addressed as well as the meta-analysis rather than report small retrospective cohorts.
Authors’ response: The authors have once again done an extensive literature search using the search terms: HIE or hypoxic ischaemic encephalopathy or perinatal asphyxia and newborn and LMIC or low and middle income countries and RCT or systematic reviews

Majority of the articles found were not addressing our focus. Our research focused on the trend in incidence and predictors of mortality from HIE in our center, which is in a lower middle income country. Majority of the RCT and systematic reviews were looking at the treatment and interventions given for HIE making it difficult to cite many of them in our study. (Since that was not our focus). We have further reviewed and cited 3 more literatures (one from Uganda [reference 5], Brazil [reference 9] and United Kingdom [reference 7])

We would be happy to review the suitability of any literature specifically pointed out or listed by the reviewer.

**Reviewer #3 comment:** Methods
There are referral biases so the incidence is calculated per neonatal admission. The Methods and Discussion must address this well. Were there changes in referral patterns? Did the proportion of admitted babies compared to other 2 NICUs in Lagos differ? I think the best solution is to acknowledge in both the Methods and Discussion (including the Conclusions) that referral bias was possible. If it can be quantified, it would be best, but it is understood that this may be hard to quantify.

Authors’ response: Our study has utilized “per admission” as the denominator of the rates in our study. Our data reports the pattern from our centre. We agree that the rates may be affected by the referral pattern. We do not have data on the admission pattern from the other 2 NICUs in Lagos. We have included this as a limitation of the study and stated it as: “Our study was a hospital -based study as opposed to a population-based study that is the gold standard for calculating incidence or prevalence of disease. There may also be referral bias among our cohort of subjects”- see line 454-456 under limitation.

**Reviewer #3 comment:** The statistical analysis must be carefully performed as referral bias can have a big impact.
Authors response: Although there are limitations with retrospective data, however, we have utilized robust statistical methods in this study. For the trends analysis, we reported rates per admission at our hospital and we went on to conduct multivariable Cox proportional analysis to correct for confounding variables.

**Reviewer #3 comment:** Results
It would be best to compare inborn and outborn infants in Tables 1 and 2

**Authors’ response:** The focus of the paper is to evaluate the predictors of mortality. “Outborn vs inborn” is one of the explanatory variables for the predictors of mortality. Outborn vs inborn is not an outcome variable in this study. Thus, the association of outborn / inborn and the major outcome was assessed in all the relevant Tables. Comparing inborn vs outborn in Tables 1 and 2 in this manuscript as suggested will change the focus of the paper as our outcome is mortality and morbidity (severity) of HIE among our cohort. Whereas, inborn/outborn is one of the explanatory variables that was assessed. The variable was also part of the multivariable model. For this manuscript to remain focused and not be unwieldy, we suggest we maintain the analysis and Tables.

**Reviewer #3 comment:** Figures 1a and 1b are not needed. Fig 1a does not have the n/N data that should be provided better in the text. A three d pie does not help.

**Authors’ response:**  Although, we believe figures 1a and 1b will visually depict the pattern of the major outcome for the audience to appreciate the burden, and our texts complimented the tables however, in line with the reviewer’s comment, Fig 1a and 1b have been expunged.

**Reviewer #3 comment:** Discussion
This study has many limitations which should be addressed but most importantly, interpret the results based on those limitations. The Conclusions miss the point totally.

Authors’ response: The limitations of this study has been extensively discussed and the fact that this study was based on single hospital data has been included in all sections of the manuscript. (See line 448 “Limitation and strength of the study”) The concluding statements have been revised and modified to capture these concerns.

**Reviewer #3 comment:** Minor
HIE and asphyxia (e.g. Figures 2a and 2c) are used. It is best to stick to one only.
The title of Figure 2c has a typo (severtly).

Authors’ response: Thank you for these observations. ‘Asphyxia’ has been changed to HIE and the typo corrected.

Once again, we are grateful to the reviewers and editors for thorough review of the manuscript. The comments have greatly improved the manuscript.

Thank you
